# MALAT1: A Pivotal lncRNA in the Phenotypic Switch of Gastric Smooth Muscle Cells *via* the Targeting of the miR-449a/DLL1 Axis in Diabetic Gastroparesis

**DOI:** 10.3389/fphar.2021.719581

**Published:** 2021-07-27

**Authors:** Yanjuan Wang, Yan Wang, Boqian Zhu, Ying Zhu, Ya Jiang, Wenjie Xiong, Lin Lin, Yaoyao Gong

**Affiliations:** ^1^ Department of Gastroenterology, The First Affiliated Hospital of Nanjing Medical University, Nanjing, China; ^2^ Department of Cardiology, Affiliated Hospital of Nanjing University of Chinese Medicine, Nanjing, China; ^3^ Department of Gastroenterology, Northern Jiangsu People’s Hospital, Yangzhou, China

**Keywords:** gastroparesis, myocytes, smooth muscle, RNA, long noncoding, diabetes, phenotypic switch

## Abstract

Diabetic gastroparesis (DGP) is a common complication of diabetes mellitus (DM). Our previous study suggested that the expression of the long non-coding RNA (lncRNA) metastasis-associated lung adenocarcinoma transcript 1 (MALAT1) is closely related to DGP. However, the role of MALAT1 in DGP pathogenesis remains unclear. Here, we aim to characterize the role of MALAT1 in DGP. First, we analyzed the lncRNA expression profiles through lncRNA sequencing. Next, we detected MALAT1 expression in the stomach tissues of DGP model mice and diabetic patients. Then, we investigated the role and mechanisms of MALAT1 in the proliferation, migration, phenotypic switch, and carbachol-induced intracellular Ca^2+^ changes in human gastric smooth muscle cells (HGSMCs) under high glucose (HG) conditions, using short hairpin RNA technology, RNA immunoprecipitation, and dual-luciferase reporter assays. We show that MALAT1 expression was upregulated in the gastric tissues of DGP model mice, the adjacent healthy tissues collected from diabetic gastric cancer patients with DGP symptoms, and in HGSMCs cultured under HG conditions. Functionally, MALAT1 knockdown *in vitro* impacted the viability, proliferation, migration and promoted the phenotypic switch of HGSMCs under HG conditions. Additionally, we show that MALAT1 sponged miR-449a, regulating Delta-like ligand 1 (DLL1) expression in HGSMCs; any disturbance of the MALAT1/miR-449a/DLL1 pathway affects the proliferation, migration, phenotypic switch, and carbachol-induced Ca^2+^ transient signals in HGSMCs under HG conditions. Collectively, our data highlight a novel regulatory signaling pathway, the MALAT1/miR-449a/DLL1 axis, in the context of DGP.

## Introduction

Diabetic gastroparesis (DGP), defined as the objectively delayed gastric emptying without mechanical obstruction, is a severe complication of diabetes mellitus (DM); it causes early satiety, nausea, vomiting, bloating, heartburn, and abdominal pain, as well as significant morbidity (Vanormelingen et al., 2013; Camilleri et al., 2017). Although the exact prevalence of DGP remains unknown, gastric emptying disorders reportedly occur in approximately one-third of diabetic patients (De Block et al., 2006; Bharucha et al., 2019). Notably, more than to affect the quality of life, DGP significantly impacts the patients’ self-management of diabetes (Kumar et al., 2018). Of note, not only hyperglycemia but also immune disorders, hormonal disturbances, autonomic neuropathy, autonomic or enteric neuropathy, gastric smooth muscle lesions, and injury of the interstitial cells of Cajal (ICCs) may result in DGP (Camilleri et al., 2011; Bharucha et al., 2019). Previous studies have demonstrated that patients with DGP show the thickening of the basal lamina around smooth muscle cells (SMCs), together with smooth muscle degeneration and fibrosis, suggesting that gastric smooth muscle lesions play a pivotal role in DGP formation (Kashyap and Farrugia 2010; Camilleri et al., 2011; Faussone-Pellegrini et al., 2012). However, the mechanisms underlying the formation of smooth muscle lesions remain unclear.

Normal gastric emptying is coordinated by SMCs, nerve cells, ICCs, glial cells, and hormones (Horvath et al., 2014). Of note, SMCs show plasticity, characterized by the reversible phenotype switch from a contractile (differentiated) to synthetic (dedifferentiated) phenotype when stimulated by some agents, such as inflammatory cytokines (Nair et al., 2011; Grover et al., 2011; Scirocco et al., 2016). This process is associated with a reduced expression of contractile markers, such as SM myosin heavy chain (MHC), calponin, α-SMA, SM22α, and smoothelin (Scirocco et al., 2016).Although this process has been more thoroughly studied in vascular SMCs, recent studies have found the plasticity of SMCs also exists in the gastrointestinal (GI) tract (Nair et al., 2011; Scirocco et al., 2016). Grover and colleagues found that SMC contractile protein smoothelin expression decreased in the gastric tissue of patients with DGP (Grover et al., 2011). Our laboratory has also reported that the expression of SMC contractile proteins was markedly reduced in the stomach tissues of a DM rat model (Yu et al., 2017).

Long non-coding ribonucleic acids (lncRNAs) are RNAs with lengths exceeding 200 bp without known protein-coding functions (Wilusz et al., 2009; Bunch, 2018). lncRNAs impact many biological processes, such as cell proliferation, migration, and differentiation (Bharucha et al., 2015; Gong et al., 2018). However, few studies have evaluated the function of lncRNAs in the pathogenesis of GI motility disorders. Our previous study found that the levels of metastasis-associated lung adenocarcinoma transcript 1 (MALAT1), a lncRNA located at the human chromosome 11q13, were elevated in the gastric tissues of patients with DM and DGP symptoms (Gong et al., 2018). Besides, we found that MALAT1 may be involved in the pathogenesis of DGP, impacting the SMC phenotypic transition (Gong et al., 2018). However, the underlying mechanisms of MALAT1 in the context of the SMC phenotypic transition and the pathogenesis of DGP remain largely unclear.

This study aimed to investigate the role of the lncRNA MALAT1 in the pathogenesis of DGP and the specific mechanism by which MALAT1 regulates the phenotypic transition of gastric SMCs. First, we detected the increased MALAT1 using lncRNA sequencing and verified that the expression of MALAT1 in the stomach tissues of a DGP mouse model and diabetic patients was indeed increased. Subsequently, we investigated the role and mechanisms of MALAT1 in human gastric SMCs (HGSMCs) under high glucose (HG) conditions, using the short hairpin RNA (shRNA) technology. Here we demonstrate that MALAT1 is upregulated under HG conditions, interacting with miR-449a, leading to the upregulation of Delta-like ligand 1 (DLL1); this ultimately leads to the phenotypic transition of SMCs and the development of DGP. Besides, as smooth muscle contraction in response to agonists is related to the increase in the intracellular Ca^2+^ concentration [Ca^2+^]_i_, we also detected the effects of the MALAT1/miR-499a/DLL1 pathway on the carbachol (CCh)-induced [Ca^2+^]_i_. Altogether, our data clarify the mechanism behind DGP, providing potential new targets for the development of novel prophylactic and therapeutic approaches in the context of DGP.

## Materials and Methods

### Tissue Specimens

All specimens were obtained from the Human Tissue Specimen Research Bank of the First Affiliated Hospital, Nanjing Medical University. Stomach specimens were collected from the adjacent normal area of the stomach of ten patients with gastric cancer and diabetes with symptoms of DGP who underwent gastrectomy. Additionally, control samples were obtained from the stomachs of ten non-diabetic gastric cancer patients with no symptoms of DGP. The collection standards for gastric specimens were as described previously (Gong et al., 2018).

### Animals and Gastric Emptying Test

Male C57BL/6J mice (6 weeks old, 20–26 g) were randomly divided into two groups: control (*n* = 10) and diabetic (*n* = 10). Diabetes was induced and diagnosed as described elsewhere (Yu et al., 2017). The solid gastric emptying experiment was performed 8 weeks after streptozotocin injection. Briefly, the mice were fasted for 16 h with free access to water and then were allowed to feed on a solid food pellet for 30 min. Then, mice were fasted again for 2 h. All mice were sacrificed at the end of fasting. The stomach was excised, and its contents were air-dried for 48 h before weighing. The gastric emptying rate was calculated using the following equation:

Gastric emptying rate(%)=[1−(dried gastric content in g)/(food intake in g)]×100

### RNA Sequencing and Analysis and Pathway and Gene Ontology Analysis

Total RNA was extracted from tissues using the Trizol reagent (Invitrogen, Carlsbad, CA, United States) and measured using the NanoDrop 1000 spectrophotometer (Thermo Fisher Scientific, Waltham, MA, United States). RNA sequencing and data analysis and pathway and gene ontology analysis was performed by Aksomics (Aksomics, Shanghai, China).

### *In vitro* Studies of Gastric Antral Contractility

Three circular muscle strips (approximately 2 mm × 10 mm) were taken from each murine gastric antral specimen and placed in Tyrode solution (37°C, 5% CO_2_ + 95% O_2_). The tension signal was recorded and analyzed using a multi-channel data acquisition and analysis system (Techman Software Co., Chengdu, China). A preload of 1 g tension was set. After tissues were equilibrated for 0.5 h, the contraction tension was recorded by the stimulation of CCh (Apexbio Technology, Houston, TX, United States). The maximum contraction tension was calculated as the maximum post-stimulation tension minus the average pre-stimulation value.

### Cell Culture

HGSMCs were cultured in Dulbecco’s modified Eagle’s medium (DMEM, Gibco, Carlsbad, CA, United States) with 10% fetal bovine serum (Gibco) and 1% penicillin-streptomycin (Hyclone, South Logan, UT, United States). All cells were cultured in an incubator with 5% CO_2_ at 37°C.

### RNA Knockdown and Cell Transfection

ShRNAs targeting MALAT1 (shMALAT1) and DLL1 (shDLL1) were designed and synthesized by GenePharma (Shanghai, China). The relevant sequences are as follows: shMALAT1-1: 5′-GCA​GCC​CGA​GAC​TTC​TGT​AAA-3’. shMALAT1-2, sense: 5′-GCC​CGA​GAC​TTC​TGT​AAA​GGA-3’. shMALAT1-3, sense: 5′-GCT​CTA​AAT​TGT​TGT​GGT​TCT-3’. shDLL1-1: 5′-GGT​ACT​GTG​ACG​AGT​GTA​TCC-3’. shDLL1-2, sense: 5′-GCT​CTT​CAC​CCT​GTT​CTA​ATG-3’. shDLL1-3, sense: 5′- GAT​GAG​TGC​GTC​ATA​GCA​ACT-3′.

The miR-449a mimic, miR-449a inhibitor, and the respective negative controls (NC) were purchased from Sangon Biotech Co., Ltd (Shanghai, China). Transfections were carried out using Lipofectamine-2000 (Invitrogen, Carlsbad, CA, United States) following the manufacturer’s instructions.

### RNA Extraction and Quantitative Real-Time Polymerase Chain Reaction

Total RNA was extracted from tissues and HGSMCs using the Trizol reagent (Invitrogen, Carlsbad, CA, United States) and measured using the NanoDrop 1000 spectrophotometer (Thermo Fisher Scientific, Waltham, MA, United States). Total RNA (1 μg) was reversely transcribed into cDNA using the PrimeScript RT Reagent Kit (Takara, Dalian, China). qPCR was performed with the SYBR Green I mix (Junxin Biotech Co., Suzhou, China) using the ABI 7500 Fast real-time PCR system (Applied Biosystems, Foster City, CA, United States). Gene expression was normalized to the expression of β-Actin or U6. Data were analyzed using the 2^−ΔΔCt^ method. The primers used in this study were all synthesized by Sangon Biotech Co., Ltd. The primer sequences used are listed in Supplementary Material (Table 1).

**TABLE 1:** Primers used in this study.

Gene	Primer	Sequence
Human MALAT1	Forward	5′- GTC​ATA​ACC​AGC​CTG​GCA​GT -3′
	Reverse	5′- CGA​AAC​ATT​GGC​ACA​CAG​CA-3′
Murine MALAT1	Forward	5′- TGC​AGT​GTG​CCA​ATG​TTT​CG -3′
	Reverse	5′- GGC​CAG​CTG​CAA​ACA​TTC​AA -3′
Human DLL1	Forward	5′- GTT​TCC​CGA​GGT​TGC​CTT​TCC -3′
	Reverse	5′- CTC​TCC​TTA​GAA​CAG​CGG​CG-3′
Murine DLL1	Forward	5′- TGA​TCG​TGG​ACA​ACA​CGG​AG -3′
	Reverse	5′- TCG​TCT​GGC​TTT​CAG​TCC​AC-3′
Human β-actin	Forward	5′- CAT​TCC​AAA​TAT​GAG​ATG​CGT​TGT -3′
	Reverse	5′- TGT​GGA​CTT​GGG​AGA​GGA​CT -3′
Murine β-actin	Forward	5′- GGC​TGT​ATT​CCC​CTC​CAT​CG -3′
	Reverse	5′- CCA​GTT​GGT​AAC​AAT​GCC​ATG​T -3′
Human U6	Forward	5′- CTC​GCT​TCG​GCA​GCA​CA-3′
	Reverse	5′- AAC​GCT​TCA​CGA​ATT​TGC​GT-3′
Murine U6	Forward	5′- ACG​GCT​ACC​TCT​CAA​TCC​CA-3′
	Reverse	5′- GAT​GCC​CGT​TGT​AGG​TCT​CC-3′
Human miR-449a	Forward	5′- GTG​CTG​TGG​CAG​TGT​ATT​GTT​A-3′
	RT	5′-GTC​GTA​TCC​AGT​GCA​GGG​TCC​GAG​GTA​TTC​GCA​CTG​GAT​ACG​ACA​CCA​GC-3′
Human miR-25-3p	Forward	5′- GCC​ATT​GCA​CTT​GTC​TCG-3′
	RT	5′-GTC​GTA​TCC​AGT​GCA​GGG​TCC​GAG​GTA​TTC​GCA​CTG​GAT​ACG​ACT​CAG​AC-3′
Human miR-34a-5p	Forward	5′- GTG​CTG​GCA​GTG​TCT​TAG​C-3′
	RT	5′-GTC​GTA​TCC​AGT​GCA​GGG​TCC​GAG​GTA​TTC​GCA​CTG​GAT​ACG​ACA​CAA​CC-3′
Human miR-429	Forward	5′- GCG​GTC​TAA​TAC​TGT​CTG​GTA​AAA​C-3′
	RT	5′-GTC​GTA​TCC​AGT​GCA​GGG​TCC​GAG​GTA​TTC​GCA​CTG​GAT​ACG​ACA​CGG​TTT-3′
Human miR-92a-3p	Forward	5′- GTC​TAT​TGC​ACT​TGT​CCC​GG-3′
	RT	5′-GTC​GTA​TCC​AGT​GCA​GGG​TCC​GAG​GTA​TTC​GCA​CTG​GAT​ACG​ACA​CAG​GC-3′
Human miR-32-5p	Forward	5′- GCG​GTG​TAT​TGC​ACA​TTA​CTA​AG-3′
	RT	5′-GTC​GTA​TCC​AGT​GCA​GGG​TCC​GAG​GTA​TTC​GCA​CTG​GAT​ACG​ACT​GCA​AC-3′
Human miR-92b-3p	Forward	5′- GTT​ATT​GCA​CTC​GTC​CCG​G-3′
	RT	5′-GTC​GTA​TCC​AGT​GCA​GGG​TCC​GAG​GTA​TTC​GCA​CTG​GAT​ACG​ACG​GAG​GC-3′
Human miR-363-3p	Forward	5′- GCG​AGC​AAT​TGC​ACG​GTA​TCC​AT-3′
	RT	5′-GTC​GTA​TCC​AGT​GCA​GGG​TCC​GAG​GTA​TTC​GCA​CTG​GAT​ACG​ACT​ACA​GA-3′

### Western Blot Analysis

Proteins were extracted from tissues and HGSMCs with the Radio-Immunoprecipitation Assay Buffer (RAPI, Junxin Biotech). Western blot analysis was carried out as described previously (Yu et al., 2017). The antibodies used were as follows: anti-α-SMA (1:1000, Ab108424, Abcam, Cambridge, United Kingdom), anti-SM22α (1:1000, D123178, Sangon Biotech Co., Ltd, Shanghai, China), anti-MHC (1:1000, Ab23990, Abcam), anti-DLL1 (1:1000, Ab10554, Abcam), anti-β-Actin (1:4000, K200058M, Solarbio, Beijing, China), HRP-conjugated goat anti-rabbit IgG (1:4000, D110058, Sangon Biotech), and HRP-conjugated goat anti-mouse IgG (1:4000, D110087, Sangon Biotech). An enhanced chemiluminescence detection kit (Junxin Biotech) was used to observe the immunoreactivity, and the Gelpro analyzer software (Infaimon S.L., Barcelona, Spain) was used for data analysis.

### Cell Viability Assay

HGSMCs were seeded into 96-well plates (4 × 10^3^ cells/well) and subjected to different treatments. Cell viability was examined with the Cell Counting Kit-8 (CCK-8, Junxin Biotech). The cells were then cultured in an incubator containing 5% CO_2_ at 37°C for 2 h. The optical density was examined at a wavelength of 450 nm using a microplate spectrophotometer (Thermo Fisher Scientific).

### Cell Proliferation Assay

HGSMCs were seeded into 48-well plates (5 × 10^4^ cells/well) and subjected to different treatments. Cell proliferation was detected using a 5-ethynyl-2′-deoxyuridine (EdU) kit (Junxin Biotech). Briefly, the cells were incubated with 200 μL EdU in a CO_2_ incubator at 37°C for 2 h. The cells were then rinsed once in cold phosphate-buffered saline (PBS), fixed with 4% paraformaldehyde, and treated with 0.5% Triton X-100. After being rinsed in PBS, the cells were incubated with carboxytetramethylrhodamine (TAMRA) and then stained with Hoechst 33,342. Stained cells were photographed using a fluorescence microscope (Olympus CKX53, Japan).

### Cell Migration Assay

Cell migration was investigated using the Transwell invasion assay (Corning, NY, United States). HGSMCs were re-suspended in 100 μL serum-free medium and then transferred into the top chamber; 600 μL DMEM containing 10% serum was added to the lower chamber. After incubation for 18 h, the cells in the upper chamber of the filter were carefully removed. Then, 4% paraformaldehyde and 0.1% crystal violet were applied to fix and stain the cells invading through the membrane, respectively. Cells were then imaged using a light microscope and counted.

### Measurement of the Intracellular Ca^2+^ Levels

The Fluo-3/AM calcium indicator (Solarbio Science & Technology Co., Beijing, China) was used to detect intracellular Ca^2+^ levels. HGSMCs were seeded into glass-bottom dishes and subjected to different treatments. Cells were then incubated with Fluo-3/AM (5 μmol/L) in a CO_2_ incubator at 37°C for 30 min and rinsed twice in PBS. Calcium fluorescence intensity was examined using a confocal laser scanning microscope (LSM510, Carl Zeiss, Oberkochen, Germany) at an excitation wavelength of 488 nm and an emission wavelength of 525 nm. The changes in Ca^2+^ fluorescence intensity are expressed as F/F0, where F0 is the basal fluorescence intensity before stimulation. CCh was used as a stimulant to generate Ca^2+^ transient signals.

### Luciferase Reporter Assays

To investigate the relationship between the lncRNA MALAT1 or DLL1 and miR-449a, we used dual-Luciferase reporter assays. The respective amplified DNA sequences were inserted into the psiCheck2 luciferase reporter vector to form the MALAT1 luciferase vector (Luciferase-MALAT1-wt), the mutated MALAT1 luciferase vector (Luciferase-MALAT1-mut), the DLL1 3′ UTR luciferase vector (Luciferase-DLL1-wt), and the mutated DLL1 3′ UTR luciferase vector (Luciferase-DLL1-mut). Then, to detect the effect of MALAT1 on miR-449a, HGSMCs were co-transfected with Luciferase-NC, Luciferase-MALAT1-wt, or Luciferase-MALAT1-mut together with the miR-449a mimic or the respective NC using Lipofectamine-2000 (Invitrogen) according to the manufacturer’s instructions. Moreover, to detect the effect of miR-449a on DLL1, cells were co-transfected with Luciferase-NC, Luciferase-DLL1-wt, or Luciferase-DLL1-mut together with the miR-449a mimic or the respective NC. 48 h after transfection, the luciferase activity was tested using the Dual-Luciferase Assay System (Promega, Madison, WI, United States) according to the manufacturer’s protocol.

### RNA Immunoprecipitation

According to the manufacturer’s instructions, RIP was carried out using the Magna RIP Kit (Millipore, Bedford, MA, United States). In brief, HGSMCs under different treatments were harvested and lysed. Then, the lysates were incubated with an anti-Argonaute two antibody (AGO2, Ab32381, Abcam) or control IgG (Ab172730, Abcam) and protein A/G beads (Biolinkedin, Shanghai, China). qPCR was used to examine the enrichment of MALAT1, miR-449a, and DLL1.

### Statistical Analysis

All experimental data are presented as the mean ± standard deviation (SD) from at least three independent experiments. Statistical differences were evaluated using the ANOVA or Student’s *t* test; the SPSS 11.0 software was used. *p* values <0.05 were considered statistically significant.

## Results

### Metastasis-Associated Lung Adenocarcinoma Transcript 1 Is Highly Expressed in Both Clinical Samples and Diabetic Gastroparesis Model Mice

**FIGURE 1:**
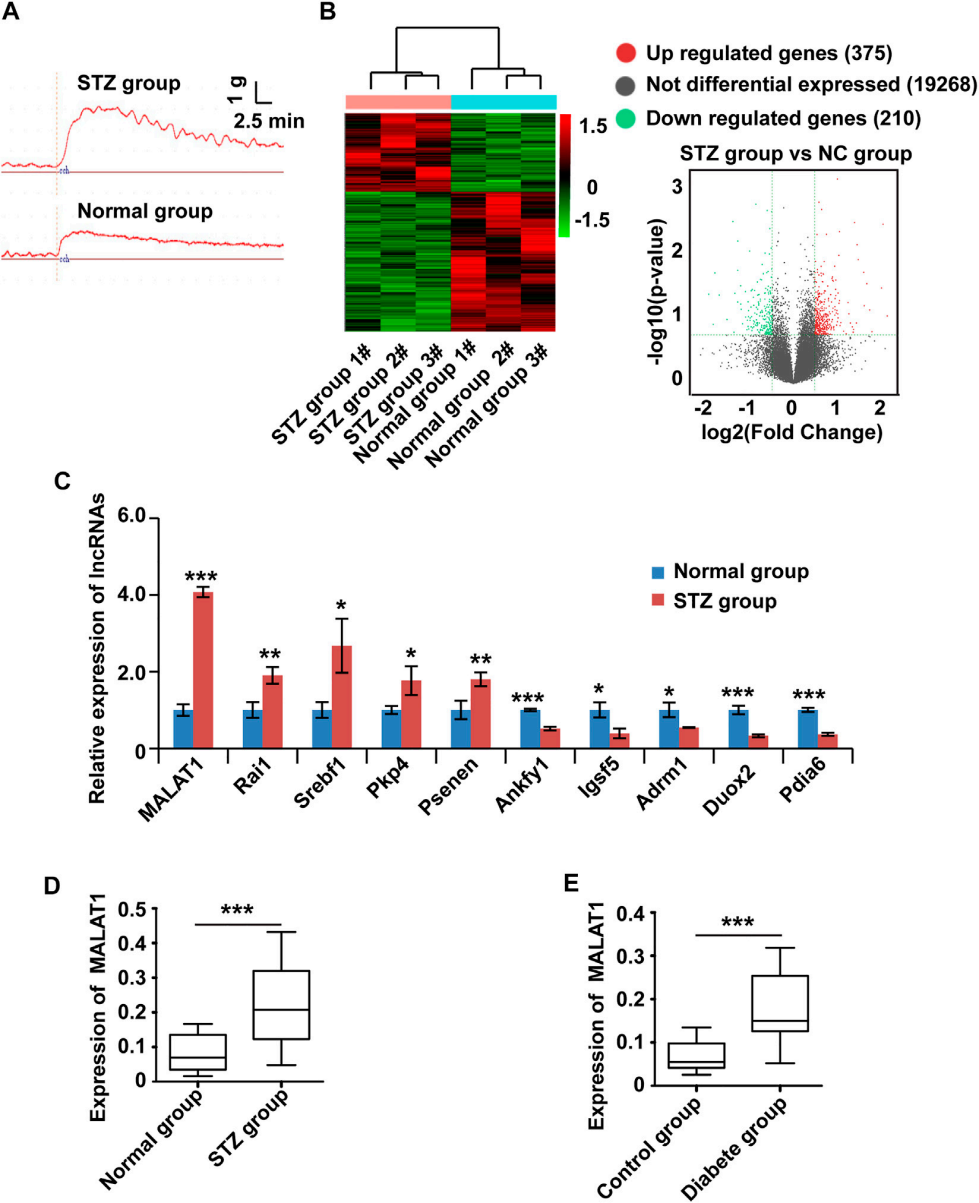
The expression of MALAT1 is upregulated in clinical samples and a murine model of DGP. **(A)** Contractility of circular gastric muscle strips from streptozotocin-induced diabetic and respective control mice after administration of 100 μmol/L CCh. **(B)** The hierarchical clustering and volcano analysis of differentially expressed lncRNAs. Here, 585 differentially expressed lncRNAs (210 downregulated and 375 upregulated) from hierarchical clustering were identified between DGP mice and the respective control group. The expression level of lncRNA is represented in red if it is above the average value of the gene in all samples. Conversely, green indicates that the expression level of lncRNA is below the average value. **(C)** qPCR was used to validate significantly differentially expressed lncRNAs, and lncRNA MALAT1 was significantly up-regulated in DGP mice. **(D)** qPCR analysis of the expression of MALAT1 in gastric tissues from a DGP mouse model (and control mice; *n* = 10 per group). **(E)** qPCR analysis of the expression of MALAT1 in (adjacent) normal stomach tissues from patients who underwent gastrectomy with or without DM and DGP symptoms (*n* = 10 per group). β-Actin was used as an internal control. ***p < 0.001, **p < 0.01, *p < 0.05.

To systematically screen the functional lncRNAs in DGP, we established a mouse model of DGP. Notably, the blood glucose levels, gastric emptying profiles, and gastric muscle strip contractility of the diabetic and control mice were measured (Supplementary Material Table 2). The gastric emptying rate and muscle strip contractility (Figure 1A) of the mice in the diabetic group were significantly lower than those of mice in the control group.

**TABLE 2:** Comparison of fasting blood glucose, gastric emptying rate and gastric muscle strip contractility in STZ group and normal group.

Group	*N*	Fasting blood glucose (mmol/L)	Gastric emptying rate (%)	Muscle strip contractility (g)
Control group	10	6.231 ± 0.725	71.111 ± 6.333	1.829 ± 1.316
STZ group	10	20.806 ± 3.339**	42.778 ± 3.993**	0.650 ± 0.145**

**p < 0.01.

Then, lncRNA sequencing was conducted in three pairs of gastric tissues from DGP mice and the respective control group to investigate the profile of lncRNA expression. Figure 1B shows the hierarchical cluster and volcano analysis of the expression profile of lncRNA. Compared with the control group, a total of 585 lncRNAs (210 down-regulated and 375 upregulated) were differently expressed in DGP tissues (fold change >2.0 and *p* < 0.05), indicating that these lncRNAs may be involved in the development of DGP. In order to verify the reliability of the sequencing results, top ten upregulated and down-regulated candidate lncRNAs were selected for qPCR, and the results were consistent with those of the sequencing analysis (Figure 1C). Next, we quantified MALAT1 expression using qPCR in the DGP mice and respective control groups. The qPCR results showed that the expression of MALAT1 in mice with DGP was significantly increased compared with that in control mice (*p* < 0.01; Figure 1D). Therefore, we focused on lncRNA MALAT1 further in the study.

To further determine the role of MALAT1 in DGP, we obtained the adjacent normal tissues from the stomach of diabetic gastric cancer patients with DGP symptoms and normal tissues from the stomach of non-diabetic gastric cancer patients without DGP symptoms. Interestingly, the expression of MALAT1 was significantly upregulated in samples from diabetic patients with DGP symptoms compared to in control individuals (*p* < 0.01; Figure 1E).

Altogether, these results indicate that MALAT1 is upregulated in the context of DGP.

### Metastasis-Associated Lung Adenocarcinoma Transcript 1 Knockdown Reverses the Phenotypic Switching of Human Gastric SMCs Under High Glucose Conditions

**FIGURE 2:**
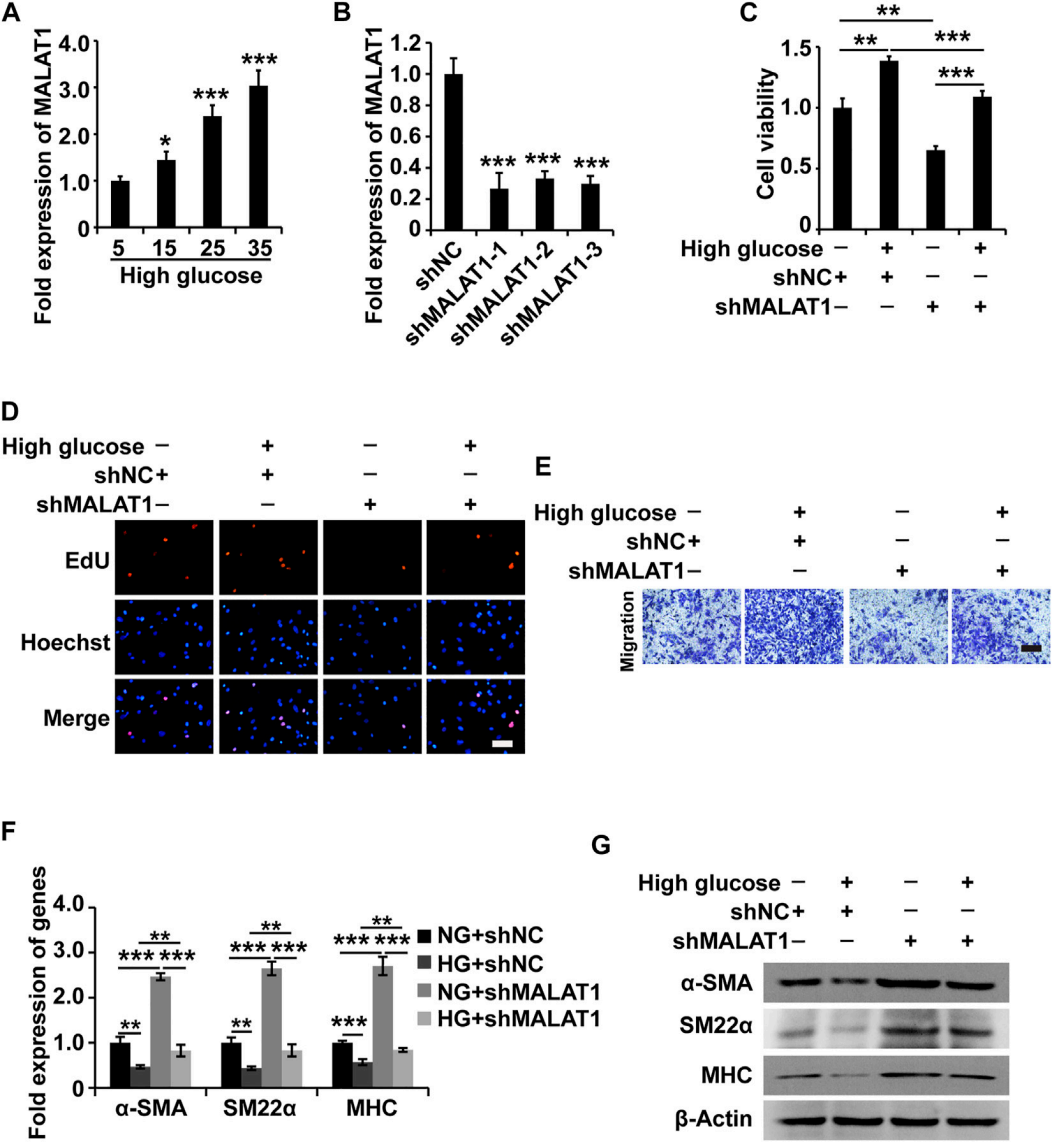
MALAT1 knockdown attenuates the HG-induced promotion of cell viability, proliferation, migration, and phenotypic switching in HGSMCs. **(A)** qPCR analysis of the expression of MALAT1 in HGSMCs treated with different concentrations of glucose (5, 15, 25, and 35 mM) for 48 h. **(B)** qPCR analysis of the expression of MALAT1 in HGSMCs 48 h after transfection with shMALAT1s and shNC. **(C–E)** The CCK8, EdU, and Transwell assays were carried out to analyze the viability, proliferation, and migration of HGSMCs under different treatments. **(F–G)** qPCR and western blot analyses of SMC contractile markers (α-SMA, SM22α, and MHC) in HGSMCs under different treatments. For **(C–G)**, four groups of cells were used: NG + shNC group, HG + shNC group, NG + shMALAT1 group, and HG + shMALAT1 group. β-Actin was used as an internal control. ***p < 0.001, **p < 0.01, *p < 0.05.

Based on the above experimental results, we hypothesized that MALAT1 might modulate the function of HGSMCs. To test this hypothesis, we examined MALAT1 expression in HGSMCs under HG conditions; qPCR results showed that MALAT1 was upregulated in HGSMCs under HG (Figure 2A). Then, we designed and synthesized three shRNAs specifically targeting MALAT1 (shMALAT1) and transfected them along with the respective control RNA (shNC) into HGSMCs. 48 h later, qPCR analysis showed that all three shMALAT1 could effectively downregulate MALAT1 expression in HGSMCs; shMALAT1-1 was selected for subsequent experiments (Figure 2B).

Then, four groups of HGSMCs were generated and analyzed: normal glucose (NG)+shNC group, high glucose (HG)+shNC group, NG + shMALAT1 group, and HG + shMALAT1 group. The CCK-8 assay results indicated that MALAT1 silencing reduced cell viability and prevented the effect of HG in the context of HGSMCs (Figure 2C). Furthermore, EdU analysis results revealed that MALAT1 knockdown inhibited cell proliferation and attenuated the HG-induced elevation of cell proliferation in HGSMCs (Figure 2D). Transwell assay results showed that MALAT1 knockdown suppressed cell migration and alleviated the HG-induced promotion of cell migration in HGSMCs (Figure 2E).

Next, we examined contractile markers’ expression to define the relationship between MALAT1 and the phenotypic switching in HGSMCs. The qPCR and western blot analyses demonstrated that MALAT1 knockdown increased the expression of the contractile markers α-SMA, SM22α, and MHC at both the RNA and protein levels, alleviating the HG-induced reduction of the expression of these genes in HGSMCs (Figures 2F,G).

In summary, these observations suggest that the silencing of MALAT1 affects the HG-induced promotion of cell viability, proliferation, migration, and phenotype switching in HGSMCs.

### Metastasis-Associated Lung Adenocarcinoma Transcript 1 Interacts With miR-449a in Human Gastric SMCs

**FIGURE 3:**
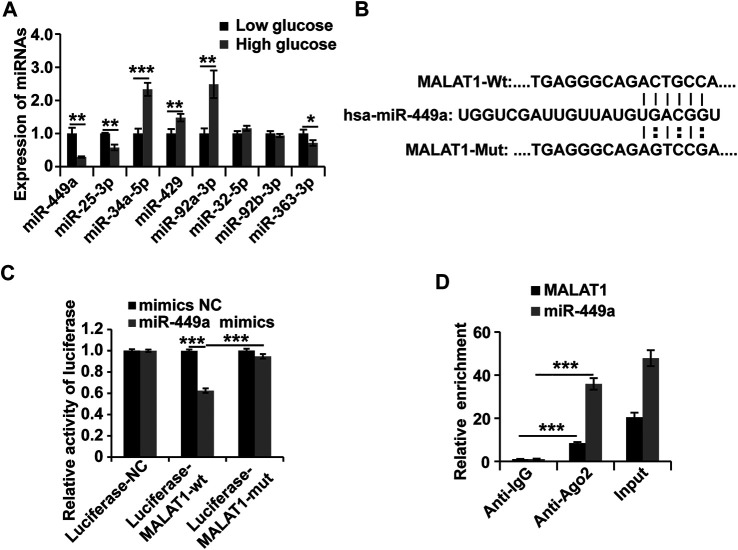
MALAT1 interacts with miR-449a in HGSMCs. **(A)** qPCR analysis was conducted to detect the endogenous microRNAs associated with MALAT1 in HGSMCs under HG conditions for about 48 h. **(B)** Predicted binding site of miR-449a in the MALAT1 sequence. **(C)** luciferase reporter assay analysis of the interaction between MALAT1 and miR-449a. **(D)** RIP assay analysis of the interaction between MALAT1 and miR-449a. U6 was used as an internal control. ***p < 0.001, **p < 0.01, *p < 0.05.

To further investigate the molecular mechanisms of MALAT1 in HGSMCs under HG conditions, we utilized the online miRcode database (http://www.mircode.org) to screen for miRNAs with MALAT1 sequence complementarity. We found that miR-449a, miR-25-3p, miR-34a-5p, miR-429, miR-92a-3p, miR-32-5p, miR-92b-3p, and miR-363-3p were complementary with MALAT1. To filter out functional miRNAs in HGSMCs under HG, we performed qPCR and observed that the expression of miR-449a was downregulated in HGSMCs under HG conditions (Figure 3A). Then, we cloned the MALAT1 cDNA containing the intact binding site of miR-449a and the MALAT1 cDNA with a mutated binding site into luciferase reporter plasmids (Luciferase-MALAT1-wt, and Luciferase-MALAT1-mut, respectively). Luciferase-MALAT1-wt, Luciferase-MALAT1-mut, and the respective control plasmids (Luciferase-NC) were transfected into HGSMCs together with a miR-449a mimic or the respective mimic NC, and luciferase activities were detected. Remarkably luciferase activity of cells transfected with the Luciferase-MALAT1-wt plasmid was significantly decreased by miR-449a mimic, while no effect occurred in Luciferase-NC- or Luciferase-MALAT1-mut-transfected cells, suggesting an interaction between MALAT1 and miR-449a (Figures 3B,C). The RIP analysis also revealed that endogenous MALAT1 pulled-down by AGO2 was specifically enriched in miR-449a-transfected cells, further confirming the interaction between MALAT1 and miR-449a (Figure 3D). Taken together, these results prove that MALAT1 directly targets miR-449a.

### Delta-Like Ligand 1 is a Target of Metastasis-Associated Lung Adenocarcinoma Transcript 1 *via* miR-449a

**FIGURE 4:**
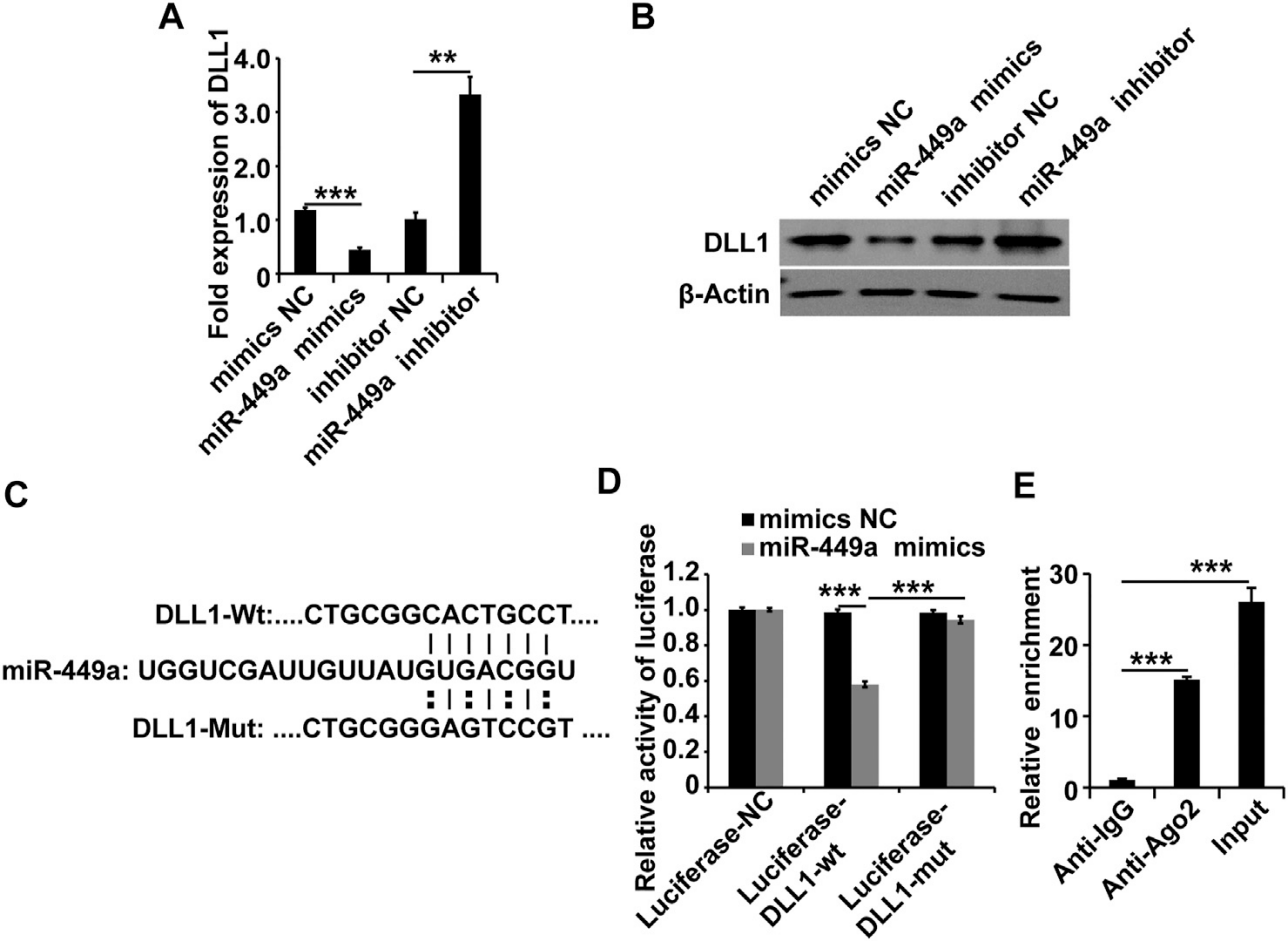
miR-449a targets DLL1. **(A–B)** HGSMCs were transfected with a miR-449a mimic, a miR-449a inhibitor, or the respective controls (NC), and 48 h later, the DLL1 mRNA and protein levels were detected by qPCR and western blotting. **(C)** Predicted binding site of miR-449a on the 3′ UTR of DLL1. **(D)** luciferase reporter assay analysis of the interaction between miR-449a and DLL1. **(E)** RIP assay analysis of the interaction between miR-449a and DLL1. U6 or β-Actin was used as an internal control. ***p < 0.001, **p < 0.01, *p < 0.05.

To explore the target of the MALAT1-miR-449a complex, the online miRNA target-prediction tool TargetScan was used. The *in silico* analysis revealed that the DLL1 3′ UTR contains a binding site for miR-449a. We transfected a miR-449a mimic, a miR-449a inhibitor, and the respective controls into HGSMCs to validate this result. The results of qPCR and western blot analyses 48 h later revealed that the miR-449a mimic inhibited the expression of DLL1 and, conversely, the miR-449a inhibitor promoted the expression of DLL1 in HGSMCs (Figures 4A,B). Furthermore, we cloned the DLL1 3′ UTR containing the exact binding site of miR-449a as well as the DLL1 3′ UTR with a mutated sequence into luciferase reporter plasmids (Luciferase-DLL1-wt, and Luciferase-DLL1-mut, respectively) and co-transfected them with the miR-449a mimic into HGSMCs. Luciferase activity was inhibited by miR-449a mimic in the context of Luciferase-DLL1-wt and restored in Luciferase-DLL1-mut-transfected cells (Figures 4C,D). A RIP analysis further proved the interaction between miR-449a and DLL1 (Figure 4E). Thus, we conclude that DLL1 is a direct target of miR-449a in HGSMCs.

### Delta-Like Ligand 1 Knockdown Reverses the Phenotypic Switching of Human Gastric SMCs Under High Glucose Conditions

**FIGURE 5:**
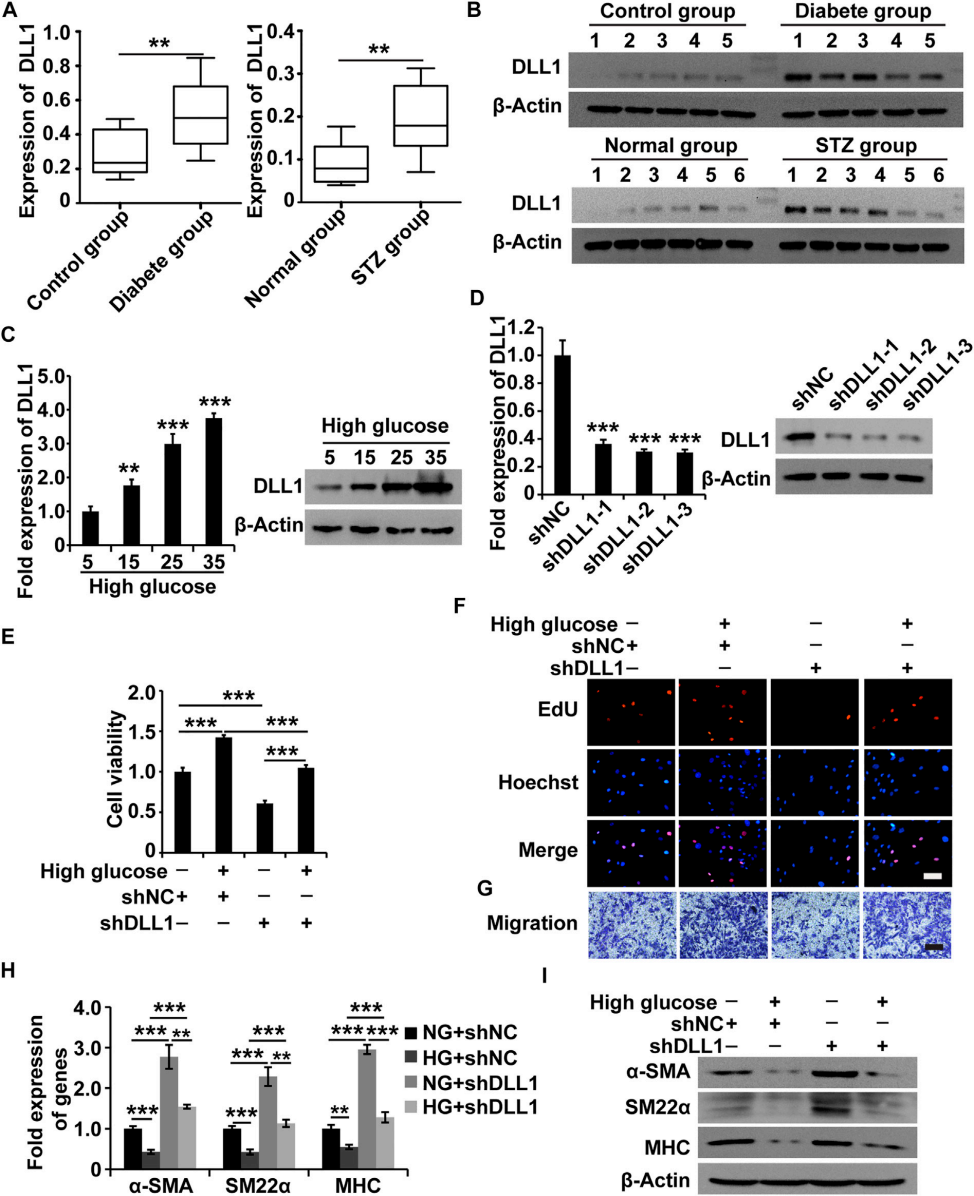
DLL1 is a target of MALAT1 *via* miR-449a. **(A)** qPCR analysis of DLL1 expressions in the adjacent normal tissues from the stomach of patients who underwent gastrectomy with or without DM and DGP symptoms (*n* = 10 per group) as well as in the gastric tissues from DGP and control mice (*n* = 10 per group). **(B)** Western blot analysis shows that the expression levels of DLL1 proteins were significantly upregulated in the gastric tissues of DGP clinical samples and DGP model mice. **(C)** qPCR and western blot analyses of the expression of DLL1 in HGSMCs treated with different concentrations of glucose (5, 15, 25, and 35 mM) for 48 h. **(D)** qPCR and western blot analyses of the expression of DLL1 in HGSMCs transfected with shDLL1s and shNC. **(E–G)** CCK8, EdU, and Transwell assays to analyze the viability, proliferation, and migration of HGSMCs under different treatments. **(H–I)** qPCR and western blot analyses of SMC contractile markers (α-SMA, SM22α, and MHC) in HGSMCs under different treatments. For **(E–I)** four groups were analyzed: NG + shNC group, HG + shNC group, NG + shDLL1 group, and HG + shDLL1 group. β-Actin was used as an internal control. ***p < 0.001, **p < 0.01, *p < 0.05.

To understand the role of DLL1 in DGP, we first measured the expression of DLL1 in the clinical samples, DGP model mice, and in HGSMCs under HG conditions. The qPCR and western blot results showed that DLL1 was upregulated in both the clinical samples and the DGP mouse model (Figures 5A,B). Furthermore, the results of qPCR and western blot analyses showed that the expression of DLL1 was increased in HGSMCs under HG conditions (Figure 5C).

Next, we designed and synthesized three shRNAs specific to DLL1 (shDLL1s) and transfected them, along with the respective control RNA (shNC), into HGSMCs. The qPCR analysis 48 h after revealed that all three shDLL1s could sufficiently downregulate the expression of DLL1 in HGSMCs; shDLL1-2 was chosen for subsequent experiments (Figure 5D).

Four groups of HGSMCs were generated and analyzed: NG + shNC, HG + shNC, NG + shDLL1, and HG + shDLL1. The results of the CCK-8, EdU, and Transwell assays showed that DLL1 knockdown led to a reduction in the cell viability, proliferation, and migration and the reversion of the HG-induced effects in HGSMCs (Figures 5E–G). Furthermore, the results of qPCR and western blot analyses revealed that DLL1 knockout suppressed the HG-induced phenotype switching in HGSMCs (Figures 5H,I). These results collectively show that the inhibition of DLL1 abrogates the impact of HG conditions on the viability, proliferation, migration, and phenotype switching of HGSMCs.

### The Metastasis-Associated Lung Adenocarcinoma Transcript 1/miR-449a/Delta-Like Ligand 1 Pathway Regulates Human Gastric SMCs Under High Glucose Conditions

**FIGURE 6:**
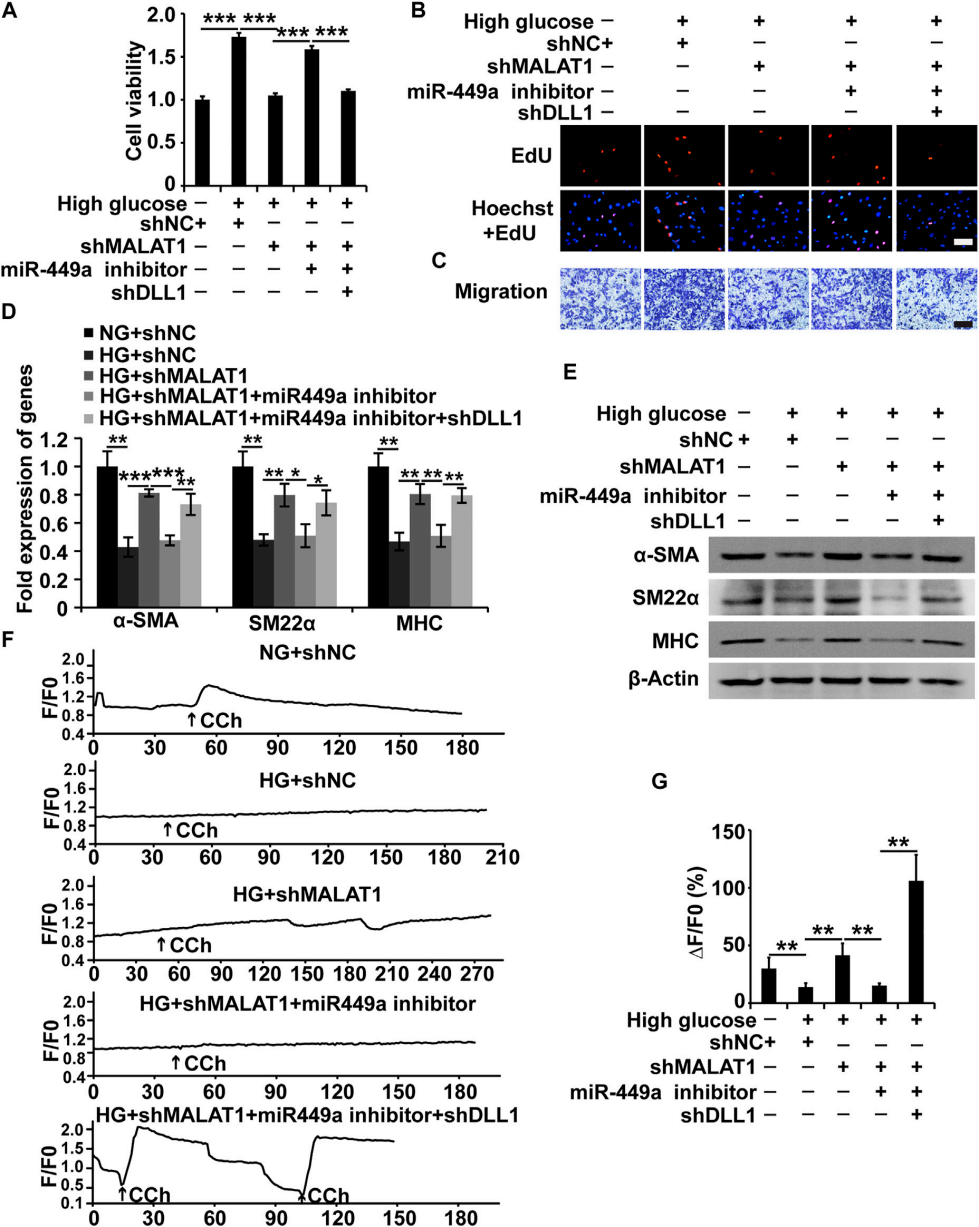
The MALAT1/miR-499a/DLL1 pathway regulates the cellular processes, phenotypic switch and CCh-induced Ca2+ transient signals in HGSMCs under HG conditions. **(A–C)** CCK8, EdU, and Transwell assays for the analysis of the viability, proliferation, and migration of HGSMCs under different treatments. **(D–E)** qPCR and western blot analyses of SMC contractile markers (α-SMA, SM22α, and MHC) in HGSMCs under different treatments. **(F)** CCh (100 μmol/L)—induced [Ca^2+^]_i_ changes in HGSMCs under different treatments. Changes in Ca^2+^ fluorescence intensity are expressed as F/F0, where F0 is the basal fluorescence intensity before stimulation. **(G)** Histogram analysis of the [Ca^2+^]_i_ changes, where ΔF is the fluorescent intensity of the response minus F0. For **(A–G)**, five groups were analyzed: NG + shNC group, HG + shNC group, HG + shMALAT1 group, HG + shMALAT1+miR-449a inhibitor, HG + shMALAT1+miR-449a inhibitor + shDLL1 group. β-Actin was used as an internal control. ***p < 0.001, **p < 0.01, *p < 0.05.

As mentioned above, we proved that MALAT1 could target miR-449a and that miR-449a could target DLL1 in HGSMCs. Additionally, we found that MALAT1 and DLL1 impacted the viability, proliferation, migration, and phenotype switching of HGSMCs. Therefore, we wondered whether MALAT1 would exert its function *via* the modulation of the miR-449a/DLL1 pathway in HGSMCs under HG conditions. To answer this question, we constructed five new groups of HGSMCs: NG + shNC, HG + shNC, HG + shMALAT1, HG + shMALAT1+miR-449a inhibitor, and HG + shMALAT1+miR-449a inhibitor + shDLL1. The CCK-8, EdU, and Transwell assays showed that the MALAT1/miR-449a/DLL1 axis modulation affected the viability, proliferation, and migration of HGSMCs under HG conditions (Figures 6A–C). Moreover, the results of qPCR and western blot analyses revealed that any disturbance of the MALAT1/miR-449a/DLL1 pathway affected the phenotype switching of HGSMCs under HG conditions (Figures 6D,E). Altogether, our data suggest that a new lncRNA-based regulatory axis is behind the pathogenesis of DGP: MALAT1/miR-449a/DLL1.

MALAT1/miR-449a/DLL1 axis effects on CCh-induced Ca^2+^ transient signals in HGSMCs.

To further demonstrate the effects of the MALAT1/miR-449a/DLL1 pathway axis on HGSMC contractility, we detected the [Ca^2+^]_i_ in the context of the above five groups under 100 μmol/L CCh treatment. As shown in Figures 6F,G, in the NG + shNC group, CCh significantly increased the mean [Ca^2+^]_i_. In the HG + shNC group, the CCh-induced Ca^2+^ response was weaker than that in the NG + shNC group. Additionally, in the HG + shMALAT1 and HG + shMALAT1+miR-449 inhibitor + shDLL1 groups, the CCh-induced [Ca^2+^]_i_ enhancement was significantly higher than that in the HG + shNC group.

## Discussion

**FIGURE 7:**
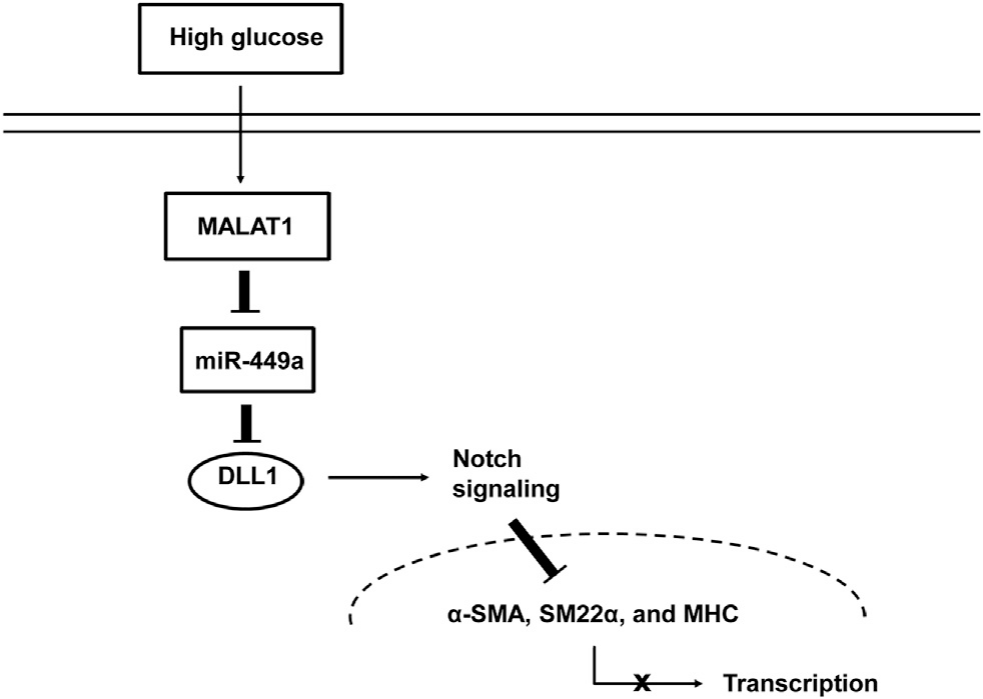
Proposed mechanism by which MALAT1 regulates the phenotypic switch of HGSMCs under HG condition.

While various studies have shown that lncRNAs are involved in the pathogenesis of many diseases, the influence of lncRNAs on GI motility-related diseases remains unclear. Our previous study (Gong et al., 2018) showed that the lncRNA MALAT1 participates in the pathogenesis of DGP. However, the roles and underlying molecular mechanisms remain elusive. Several major observations were made in this study. First, we introduced the lncRNA array to systematically screen the lncRNAs differentially expressed in the DGP mouse model and demonstrated that MALAT1 was upregulated in both DGP mice and clinical samples. Next, we proved that HG enhanced cell viability, proliferation, and migration of HGSMCs, and promoted their phenotype switching; importantly, MALAT1 knockdown reversed this phenotype. Then, we demonstrated that MALAT1 exerted its role *via* sponging miR-449a in HGSMCs. Finally, we found out that DLL1 is the downstream target of miR-449a. Thus, our research highlights a new regulatory axis (MALAT1/miR-449a/DLL1) in the context of DGP. The model diagram is shown in Figure 7.

As one of the common complications of diabetes, DGP leads to many upper GI symptoms. Of note, these symptoms can be severe and affect the patients’ glycemic control (Camilleri et al., 2017; Bharucha et al., 2015). Since the pathogenic mechanism of DGP has not been fully elucidated, there is a lack of effective treatments. It is currently believed that DGP is probably associated with neuropathy, ICC injury, smooth muscle abnormalities, acute fluctuations in blood glucose, and changes in gastrointestinal hormones (Bharucha et al., 2019; Camilleri et al., 2011). SMCs are behind the contraction process and are essential for regular gastric emptying; of note, the contractile ability of SMCs depends on a contractile phenotype (Scirocco et al., 2016; Sanders et al., 2012). As mentioned above, SMCs have intrinsic plasticity, being capable of undergoing reversible changes in phenotype, a process referred to as “phenotypic switch” under different stimuli (Scirocco et al., 2016). This phenomenon has been more thoroughly studied in vascular SMCs; in response to injury, the expression of genes related to contractility is suppressed, whereas the expression of genes related to synthetic, migratory, and proliferative functions is upregulated (Scirocco et al., 2016). However, the phenotypic switch of GI SMCs in the context of GI motility diseases and the regulatory signaling pathways behind them remains poorly investigated. Our previous study found that the expression of contractile markers markedly decreased in the gastric tissues of diabetic patients with DGP symptoms (Gong et al., 2018). This study verified that HG could cause an abnormal proliferation, migration, and phenotype switching of HGSMCs, which may lead to DGP-related smooth muscle abnormalities.

Several studies have shown that lncRNAs play an important role in the pathogenesis of diabetes and its complications (Zhang et al., 2020; He et al., 2020). We demonstrated that MALAT1 was elevated in the tissue samples from diabetic patients with DGP symptoms as well as in a murine model of DGP. Furthermore, we clarified that MALAT1 mediates the proliferation, migration, phenotypic switching, and CCh-induced [Ca^2+^]_i_ changes of HGSMCs in the context of HG conditions. LncRNAs have been proposed to function as competing endogenous RNAs, absorbing miRNAs and regulating their downstream signaling pathways (Salmena et al., 2011; Tay et al., 2014; Thomson and Dinger, 2016). In the present study, we found that MALAT1 could control the levels of miR-449a precisely by acting as a sponge. Of note, the downregulation of miR-449a promoted the expression of DLL1. Therefore, MALAT1 exerted its function *via* the miR-449a/DLL1 signaling pathway. Notably, the MALAT1/miR-449a/DLL1 regulatory axis is a novel regulatory pathway in DGP.

DLL1 is a mammalian ligand of Notch receptors; interactions between DLL1 and Notch regulate the Notch signaling pathway (Dyczynska et al., 2007). Accumulating evidence shows that the Notch signaling pathway plays a pivotal role in controlling the differentiation and phenotypic switching of vascular SMCs (Morrow et al., 2008; Liu et al., 2018). Here, we found that DLL1 is a direct target of miR-449a; of note, the inhibition of DLL1 reversed the effects of HG. We speculate that DLL1 regulates the phenotypic switching of HGSMCs under HG conditions *via* the activation of the Notch signaling pathway.

A limitation of this study was that the clinical specimens analyzed were frozen (stored at -80°C) tissues obtained from the Human Tissue Specimen Research Bank; thus, we were unable to associate pathological characteristics in patient tissue with the expression of MALAT1.

In conclusion, here we show that the expression of MALAT1 is upregulated in the gastric tissues of a DGP mouse model, the adjacent normal gastric tissues collected from diabetic gastric cancer patients with DGP symptoms and in HGSMCs cultured under HG conditions. The upregulation of MALAT1 promotes the phenotype switching of HGSMCs under HG conditions *via* the miR-449a/DLL1 axis. These findings highlight a novel regulatory signaling pathway in the context of DGP that deserves to be further characterized in the quest for new diagnostic biomarkers and treatment approaches for DGP.

## Data Availability

Data for experiment are available from the corresponding author (YG) upon reasonable request. The RNA sequencing data presented in this study can be found in online repository. The name of the repository and accession number can be found below: NCBI Sequence Read Archive PRJNA743433.
